# Cryopreserved Platelets in a Non-Toxic DMSO-Free Solution Maintain Hemostatic Function In Vitro

**DOI:** 10.3390/ijms241713097

**Published:** 2023-08-23

**Authors:** Kristina Ehn, Agneta Wikman, Michael Uhlin, Per Sandgren

**Affiliations:** 1 Department of Clinical Immunology and Transfusion Medicine (KITM), Karolinska University Hospital, 141 86 Stockholm, Sweden; agneta.wikman@regionstockholm.se (A.W.); michael.uhlin@ki.se (M.U.); per.sandgren@regionstockholm.se (P.S.); 2 Department of Clinical Science, Intervention and Technology (CLINTEC), Karolinska Institutet, 141 52 Huddinge, Sweden

**Keywords:** transfusion, platelets, cryopreservation, dimethyl sulfoxide (DMSO, Me2SO), activation, microparticles

## Abstract

Dimethyl sulfoxide (DMSO) is regularly used as a cryoprotectant agent for the cryopreservation of platelets. However, DMSO is considered toxic. We therefore hypothesized that saline could be used as a non-toxic medium for the cryopreservation of platelets. Double-dose buffy coat platelets (*n* = 10) were divided and cryopreserved at −80 °C using 5–6% dimethyl sulfoxide (DMSO) or in NaCl (9 mg/mL). Paired testing was conducted pre-freeze, post-thaw (PT 1 h). Upon analysis, each bag was thawed and reconstituted in fresh plasma. Analyses included cell counts and the metabolic, phenotypic, and functional properties of the platelets together with thromboelastometry. The cryopreserved platelets showed several biochemical and ultrastructural changes compared to pre-freezing. Platelet recovery was approximately 17% higher in DMSO-free units (*p* < 0.001), but the platelet viability was reduced (*p* < 0.001). However, using controlled freezing (*n* = 6), the platelet viability was improved. The clot formation time (CFT) was comparable, but DMSO-free platelets showed slightly decreased maximum clot firmness (MCF) (*p* = 0.034). By reducing the reconstituted plasma volume, a reduced CFT and increased MCF were obtained (*p* < 0.001). This study demonstrates that platelets can be cryopreserved in saline without the addition of DMSO, with high recovery and maintained hemostatic function. However, controlled freezing is required to optimize platelet quality.

## 1. Introduction

Platelets are key players in hemostasis, and platelet transfusions are critical in the treatment of bleeding [1]. However, many challenges concern the availability of platelets since they have a short shelf time of 5–7 days, resulting in high wastage and high costs and often limited access in remote locations. The cryopreservation of platelets was first mentioned in a paper more than 65 years ago, where the platelets were frozen at −15 °C and later transfused [2]. Since then, the method has evolved, and nowadays platelets are frozen and stored at −80 °C [3]. The original method containing dimethyl sulfoxide (DMSO, Me2SO) as a cryoprotectant agent (CPA) was developed in 1974 by Robert C. Valeri [4]. The method was further optimized by removing excess DMSO before freezing, to avoid the post-thaw wash, which enhanced platelet pre-activation [5]. This method has become the standard routine for platelet cryopreservation [6]. Previously, cryopreserved platelets were used mainly for special indications, but recently there has been increasing interest in the treatment of bleeding [7,8]. 

Although the 6% DMSO no-wash protocol is generally used, the method has disadvantages. One issue is the toxicity of DMSO. Not only does the freezing process damage the cells, but DMSO may also worsen the damage if cells are exposed for too long and not kept cool [9]. Studies have demonstrated in vitro platelet recovery of 50–70% after cryopreservation [10,11,12]. Most cells display a pre-activated phenotype and the increased release of platelet-derived microparticles [13,14,15]. Studies also confirm the downregulation of various platelet receptors, including glycoprotein Ibα (CD42b), platelet von Willebrand factor receptor, glycoprotein VI (GPVI), collagen receptor, and platelet and endothelial cell adhesion molecule 1 (PECAM-1). Cryopreservation adversely affects viability, with lower membrane potential compared to fresh platelets [10,11,12]. 

The main action for CPAs is to prevent the formation of intracellular ice crystals and protect the ultrastructures of the cells. The cell membrane is the most vital structure affected by the freezing process, as adverse changes in the lipid organization and osmotic pressure occur when the temperature shifts [16,17]. Various CPAs have been evaluated to minimize platelet lesions during freezing, most often in combination with DMSO. Alternative CPAs include propane-1,2-diol and glycerol cocktails with hydroxyethyl starch and dextran, as well as with glucose [18,19,20,21,22]. Other mixtures contain DMSO with added second messengers such as ThromboSol1 and epinephrine or recrystallization inhibitors such as N-(2-fluorophenyl)-d-gluconamide [23,24,25]. In the majority of studies, it was concluded that the cryopreserved platelets displayed similar defects as with the conventional DMSO freezing protocol [20,23,24]. Additionally, it has been shown that alternative CPAs excluding DMSO also can induce toxic side effects [18,19,21]. The most recent studies comprising trehalose or trehalose combined with phosphate have been conducted to decrease the pre-activation of the platelets [26,27]. Studies have also tried to remove DMSO prior to and post-freezing to reduce the final concentration of DMSO [28,29]. Furthermore, the usage of controlled-rate freezing (CRF) was evaluated by our group to study the risk of intracellular ice formation or dehydration during the freezing process. The study concluded that CRF was not superior to uncontrolled freezing (UCRF) when using the standard DMSO protocol [12].

Although multiple attempts to find alternative CPAs have been conducted, many of them include DMSO, and DMSO-free alternatives are complex in terms of toxic side effects and technology. Thus, in a series of pilot experiments, the effects of freezing using saline without the addition of DMSO were initially tested. Surprisingly, we observed that the addition of high-volume isotonic saline showed preservative effects on platelets after freezing and thawing, hypothetically indicating a reduction in the intracellular water since destructive ice crystallization would otherwise have occurred. The mechanisms behind this are unclear but may be linked to the prevailing discrepancy regarding osmosis in general [30]. In this study, we further show that it is possible to cryopreserve platelets without any sheltering protection of a CPA and still retain hemostatic functionality. This non-toxic approach would minimize the risk of adverse effects from DMSO, on the platelets as well as in the recipients.

## 2. Results

### 2.1. Improved Platelet Recovery in DMSO-Free Concentrates

**Figure 1:**
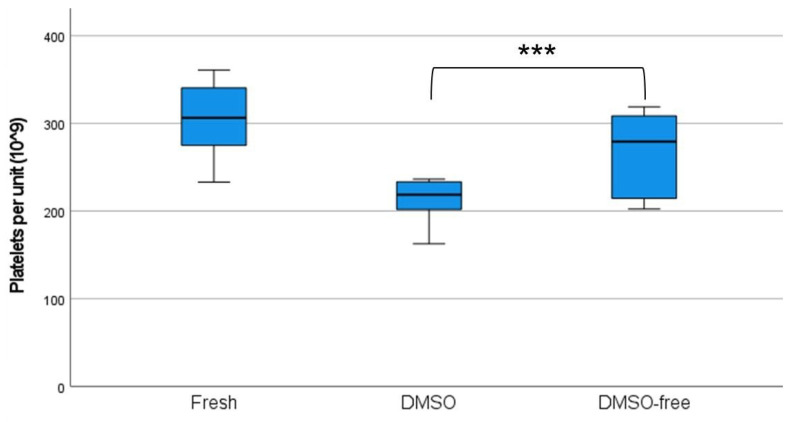
Increased platelet counts in DMSO-free concentrates. *** *p* < 0.001.

Platelets thawed after cryopreservation with the DMSO-free protocol showed platelet recovery of 87.4% ± 8.6% compared to cryopreservation with the conventional DMSO method, which displayed recovery of 69.6% ± 6.1% (*p* < 0.001). The number of platelets per unit before freezing was 306 ± 38.2 × 10^9^/unit (1445 ± 161 × 10^6^/mL). After thawing, units frozen with DMSO presented a mean of 212 ± 25.8 × 10^9^/unit (1005 ± 147 × 10^6^/mL), while DMSO-free units amounted to 267 ± 45.2 × 10^9^/unit (1236 ± 198 × 10^6^/mL) (*p* < 0.001) (Figure 1).

The number of platelets per unit was measured before and after freezing to examine the platelet loss after cryopreservation. Boxplots display a median with 95% confidence intervals (*n* = 10). A two-tailed *t*-test was performed between the DMSO and DMSO-free groups, defined as * *p* < 0.05, ** *p* < 0.01, and *** *p* < 0.001. Error bars indicate 95% CIs.

### 2.2. Changes in Extra- and Intracellular Metabolic Parameters 

Extra- and intracellular metabolic parameters were examined before and after cryopreservation with DMSO and without DMSO. Significant differences could be shown for various parameters (Table 1). DMSO-free products showed higher partial pressure oxygen (pO2), resulting in less partial pressure carbon dioxide (pCO2) in the product. Other parameters, such as pH, lactate, and bicarbonate, also showed variations. The platelet volume, glucose, and ATP generation remained comparable between the two groups (Table 1).

### 2.3. Similar Phenotypic Expression after Cryopreservation

**Figure 2:**
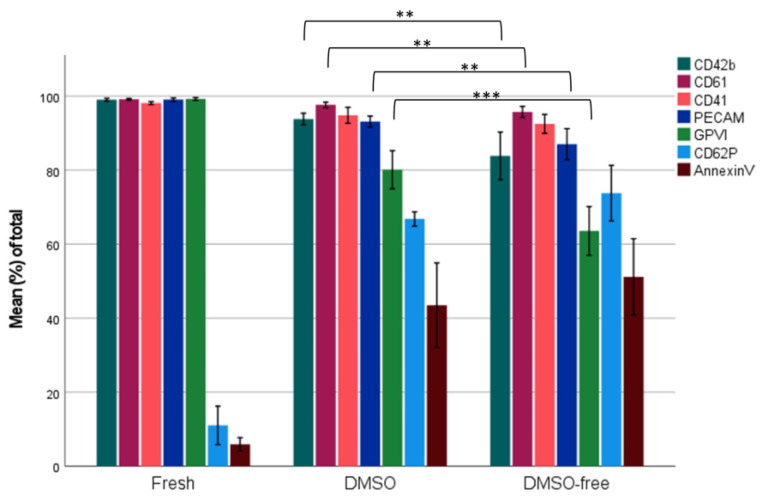
Cryopreservation of platelets alters the phenotypic expression of various platelet markers. The phenotypic expression of various platelet-specific structures was measured before and after freezing with two different cryomedia. Results from fresh platelets (double-dose buffy coat platelets pre-freezing), post-thaw DMSO, and post-thaw DMSO-free cryopreserved platelets are displayed, with bars showing the mean value of *n* = 10. A two-tailed *t*-test was performed between the DMSO and DMSO-free groups, defined as ** *p* < 0.01, and *** *p* < 0.001. Error bars: 95% CI.

The expression of various platelet receptors showed both up- and downregulation after cryopreservation. Both cryopreserved groups were more activated, with the elevated expression of P-selectin (CD62P), from a mean of 11.43 ± 5.95% of total fresh platelets to 66.81 ± 2.70% with DMSO and 73.78 ± 10.50 with DMSO-free medium. The expression of phosphatidylserine (Annexin V+) was also elevated in the cryopreserved groups, i.e., DMSO with 43.50 ± 15.98% and DMSO-free with 51.16 ± 14.40%, compared to fresh platelets with 5.93 ± 2.13%. In addition, cryopreserved platelets displayed the downregulation of receptors, with some further reduced in the DMSO-free group. Among the receptors with decreased expression were glycoproteins Ib, IIIa (CD42b, CD61), and VI and platelet endothelial cell adhesion molecule (PECAM-1) (Figure 2).

Furthermore, the response upon activation with agonist ADP and collagen was examined. No significant difference could be shown between the two study groups, neither before stimulation nor after stimulation with either ADP or collagen. However, there was a significant difference (*p* > 0.001) in the base activation (PAC-1+) of the platelets before freezing, being 6.34 ± 6.83% (fresh platelets), compared to after freezing, with DMSO yielding 49.94 ± 11.28% and DMSO-free yielding 50.09 ± 15.36%.

### 2.4. Increased Release of Platelet-Derived Microparticles in Cryopreserved Concentrates 

The amount of platelet-derived microparticles was measured through flow cytometry according to their size (<1 µm) and the expression of platelet glycoprotein IIIa (CD61). Fresh platelets displayed a significantly lower proportion of platelet-derived microparticles, with a mean of 3.19% ± 1.20%, while cryopreserved displayed 24.78% ± 0.64% (DMSO) and 25.41% ± 2.00% (DMSO-free), respectively. Additionally, the expression of phosphatidylserine detected with Annexin V-FITC was measured to investigate the procoagulant profiles of the microparticles. No significant difference was found between the three groups (*p* = 0.056), with a mean of 4.92% ± 2.32% for fresh platelets, 4.77% ± 2.84% for DMSO, and 2.60% ± 1.55% for DMSO-free cryopreserved platelets.

### 2.5. Controlled-Rate Freezing Restores Platelet Viability in DMSO-Free Concentrates

**Figure 3:**
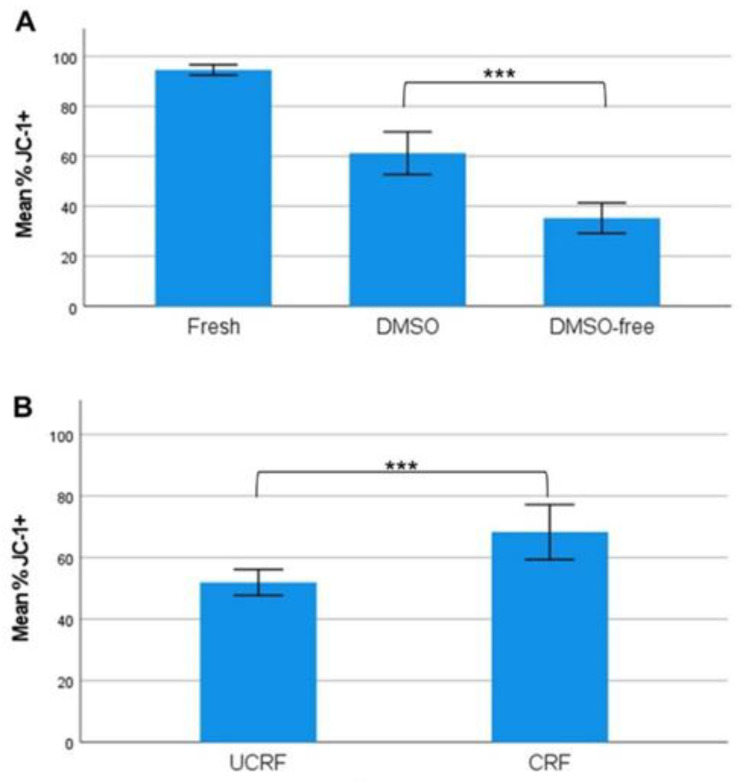
Platelet viability after DMSO and DMSO-free cryopreservation at different freezing rates. Mitochondrial membrane potential (JC-1+) was measured to estimate platelet viability in fresh, DMSO-containing, and DMSO-free cryopreserved platelets. (**A**) JC-1 expression (mean) in DMSO and DMSO-free cryopreserved platelets (*n* = 10) with 95% error bars. (**B**) JC-1 expression (mean) with 95% error bars for DMSO-free cryopreserved platelets (*n* = 6), frozen with either fast-rate uncontrolled freezing (UCRF) or controlled-rate freezing program (CRF). A two-tailed *t*-test was performed between the DMSO and DMSO-free groups, as well as between the UCRF and CRF groups, defined as *** *p* < 0.001. Error bars: 95% CI.

Platelet viability was analyzed by measuring the platelet mitochondrial membrane potential using flow cytometry. The mitochondrial membrane potential was reduced (*p* < 0.001) (*n* = 10) in DMSO-free CP, with a mean of 35.3% ± 8.5%, compared to CP with DMSO, with a mean of 61.2% ± 11.9% (Figure 3A). When controlled-rate freezing was applied in a new set of experiments (*n* = 6), the platelet viability was recovered in the DMSO-free group, from 52.03% ± 4.02% with uncontrolled-rate freezing to 68.27% ± 8.52% with controlled-rate freezing (*p* < 0.001) (Figure 3B).

### 2.6. Platelet Concentration and Freezing Rate Affect Platelet Coagulation Capacity

**Figure 4:**
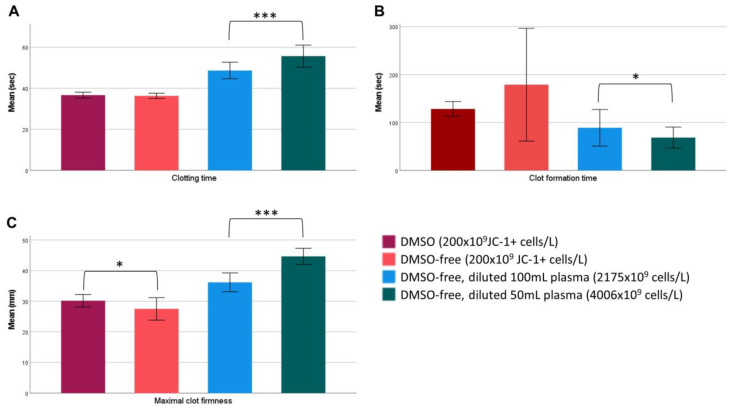
Hemostatic properties of cryopreserved platelets are affected by the platelet concentration. Thromboelastometry was performed to estimate the hemostatic function of cryopreserved platelets. Panels display (**A**) clotting time, (**B**) clot formation time, and (**C**) maximal clot firmness. DMSO-free platelets showed no significant difference compered to DMSO regarding CT and CFT, but slightly reduced maximal clot firmness. However, when the DMSO-free products were reconstituted in less plasma to a higher platelet concentration, all three parameters were affected. *p*-values are defined as * *p* < 0.05 and *** *p* < 0.001. Error bars: 95% CI.

Thromboelastometry was performed to assess the in vitro coagulation capacity of DMSO and DMSO-free CP. Fresh platelets were examined to monitor the base values, as well as the fresh frozen plasma (FFP) that the samples were diluted in. Fresh platelets (200 × 10^9^/L) (*n* = 10) showed a clotting time of 47.7 ± 2.4 s, clot formation time of 52.5 ± 5.5 s, and maximal clot firmness of 57.0 ± 4.1 mm. FFP without platelets, analyzed in duplicate, displayed a clotting time of 56 s, clot formation time of 505 s, and maximal clot firmness of 23 mm. ROTEM analyses of DMSO and DMSO-free CPs was performed according to the number of viable cells, with a loading of 200 × 10^9^ JC-1+ cells/L per test (*n* = 8). No significant difference was shown in the clotting time, (s) 37.1 ± 1.6 vs. 36.9 ± 1.5 (*p* = 0.649), or clot formation time (s), 119.9 ± 24.6 vs. 168.8 ± 102.6 (*p* = 0.175), respectively. However, the maximal clot firmness was significantly (*p* = 0.034) higher in the DMSO group, with a mean of 31.5 ± 3.5 mm, compared to the DMSO-free group, with a mean of 28.0 ± 3.6 mm (Figure 4).

In a second step, with a new set of experiments (*n* = 6), DMSO-free concentrates were frozen using CRF (mean; 1300 × 10^9^ cells/L). With CRF, the MCF increased from a mean of 33.4 mm ± 5.4 mm (UCRF) to 35.2 mm ± 6.2 mm (CRF). The CFT was reduced from 102.2 s ± 63.8 s (UCRF) to 94.2 s ± 66.9 s (CRF), but the differences for MCF and CFT were not significant.

Subsequently, in a third experiment, we aimed to test the coagulation effect by increasing the platelet concentration in DMSO-free units (*n* = 6) by reconstitution in either 100 mL (mean: 2175 × 10^9^ cells/L) or 50 mL (mean: 4006 × 10^9^ cells/L) blood-group-compatible plasma. Results showed that all three parameters were significantly affected by the added volume of plasma, indicating that the coagulation properties were improved in more concentrated platelet products. The clotting time was increased by 7.0 s (*p* < 0.001), the CFT decreased by 20.5 s (*p* = 0.031), and the MCF was increased by 8.5 mm (*p* < 0.001) when less plasma was added (Figure 4).

### 2.7. DMSO and DMSO-Free Platelets Display Similar Ultrastructural Changes through TEM Imaging

**Figure 5:**
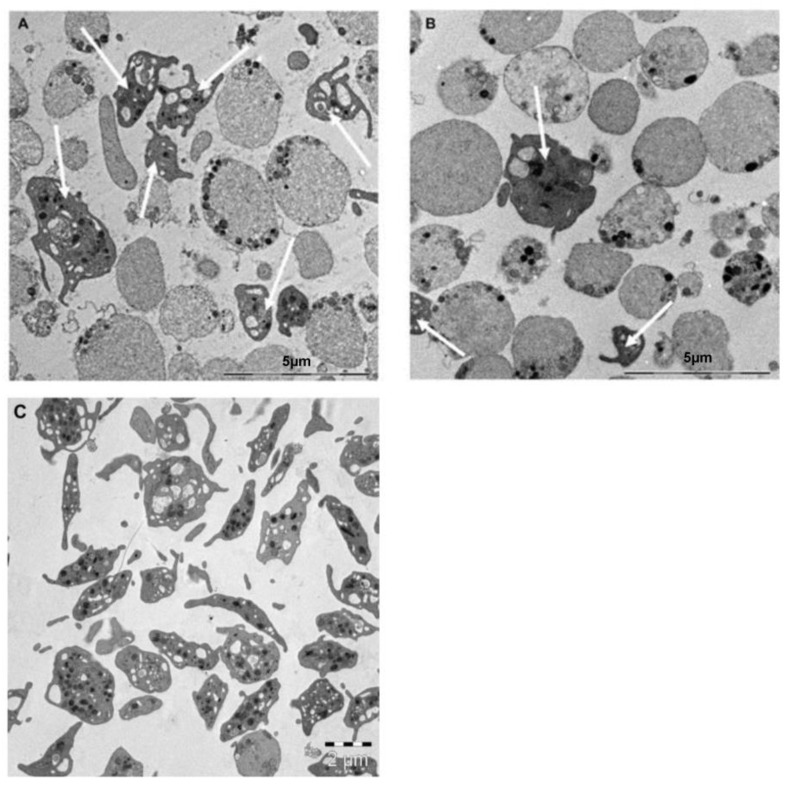
TEM images of DMSO and DMSO-free cryopreserved platelets. (**A**) DMSO-containing cryopreserved platelets, (**B**) DMSO-free cryopreserved platelets, and (**C**) fresh day-1 platelets showing normal ultrastructure, including alpha, dense granules, and glycogen in clusters. Both (**A**,**B**) demonstrate an increased number of balloon-like platelets with no or few cytoplasmic granules clustered along the cell membrane. A limited number of normal cells can be recognized by the irregular cell surface and cytoplasmic granules (pointed out with white arrows). Balloon-like platelets appear completely smooth, apart from an occasional membrane bleb. The cytoplasm of balloon-like platelets contains only a few (if any) randomly distributed organelles or remnants thereof. Such examples are equally present in both groups, including highly diluted cytoplasm and a few inclusions as well as moderately dense cytoplasm and higher levels of organelles.

Electron microscopy was performed to investigate the altered morphology of cryopreserved platelets after freezing. Both DMSO and DMSO-free platelets displayed a highly diminished morphology with a few normal cells and an increased number of rounded cells (balloon-like platelets), with cytoplasmic granules randomly distributed or polarized to the periphery of the cell (Figure 5).

## 3. Discussion

It is well known that the cryopreservation of platelets affects their characteristics and function. Efforts have been made to find alternative cryoprotectant agents (CPA) without or with less usage of DMSO [18,19,20,21,23,24,25,26,27,31]. Despite all efforts, the original method developed in the 1970s continues to be the preferred method [4,5]. Thus, the optimal approach for platelet cryopreservation remains to be explored. In this study, we explored an alternative freezing protocol by excluding DMSO and freezing the cells in saline without the addition of DMSO. We then compared our results in a paired study design with conventional cryopreserved platelets with DMSO. The results showed that DMSO-free platelets exhibited improved recovery with a higher number of platelets in the final product (87.4% ± 8.6% vs. 69.6% ± 6.1%). For conventional DMSO products, the results are in line with previous studies showing platelet recovery between 50 and 70% [8,10,11,12,32]. 

Electrolytes were examined to confirm that the salt balance was normalized after cryopreservation with a high volume of saline. Likewise, pH, lactate, and bicarbonate were all within accepted values. Notably, DMSO-free platelets showed a higher concentration of partial-pressure oxygen compared to DMSO, leading to a decreased concentration of partial-pressure carbon dioxide. This could conceivably indicate limited aerobic capacity after reconstitution. Although the ATP generation was equivalent between groups, it could not be ruled out that the reduced mitochondrial function in the DMSO-free units could be linked to the increased oxygen pressure observed. Such a situation was earlier characterized and is considered to have a negative impact on platelets with respect to conventional storage [33]. However, after thawing and reconstitution, DMSO-free frozen platelets are not intended for storage. It has previously been shown that a loss of membrane potential also plays a key role in the mechanism of procoagulant platelet formation [34], which, in the context of platelet transfusion in bleeding situations, may still be beneficial.

In line with previous studies, we confirmed that the cryopreservation of platelets leads to alterations in various receptors compared to fresh platelets [10,11,12,32]. Using saline, the expression of glycoproteins (GP) Ib, IIIa, and VI (CD42b, CD61), along with PECAM-1, was further decreased. Impaired expression of GPVI has previously been linked to mitochondrial damage [35,36,37] and a reduced collagen response [10,38]. We could observe that the DMSO-free platelets showed lower mitochondrial membrane potential (MMP). However, this study did not show any differences between groups in platelet responses to stimulation with collagen. Hypothetically, the equally high background activation level may have been caused by the freezing process itself. Cryopreserved platelets exhibit an increased amount of platelet-derived microparticles (PMPs) [13,14,15,39]. These PMPs have been shown to be procoagulant and support faster clotting in cryopreserved platelet products [13,15,40]. It is suggested that the expression of phosphatidylserine (PS) promotes coagulation by creating a catalytic surface that enhances the binding of coagulation factors, activating platelets to initiate the fibrin network and platelet aggregation [13,41,42,43]. In this study, the fraction of Annexin-V-positive PMPs was similar in all groups both before and after freezing. However, previous studies have found the fraction of Annexin-V-positive PMPs to be much higher [13,14,15]. This might be linked to a discrepancy in methodology, as we could not detect as many Annexin-V-positive PMPs. Nonetheless, we could, as in other studies, see faster clotting initiation after cryopreservation. This may be an effect of the greater quantity of PMPs, as well as the increased expression of Annexin V+ on the cryopreserved platelets.

To support our phenotypic findings, we performed TEM imaging. Through TEM imaging, we observed that DMSO and DMSO-free platelets showed similar ultrastructural changes, including swelling. The TEM images strongly illustrated a balloon-like morphology in many of the platelets after freezing and thawing, which was earlier described as a feature of procoagulant platelets [44]. Ballooning has been shown to be an irreversible process that increases the area for coagulation assembly, affects PS exposure, and releases PMPs [45].

The coagulation properties of the cryopreserved products were shown to be somewhat influenced by the CPA as DMSO-free concentrates displayed reduced maximal clot firmness. However, both the clotting time and clot formation time remained comparable. CT is mainly affected by the coagulation factors, while CFT combines the interaction of coagulation factors, platelets, and fibrinogen. The maximal clot formation measures the clot strength, which is affected by the fibrinogen concentration, factor XIII, and platelets [46,47]. The weakened stability of the clot is likely the result of platelets harmed by the freezing process. Although no significant difference could be shown regarding platelets’ responsiveness to agonists, DMSO-free platelets showed a reduced MMP together with the reduced expression (%) of platelet receptors. On one hand, this reduction might have been the cause of the weakened stability of the platelet clot as fewer platelets maintained their full aggregating capacity. On the other hand, the loss of membrane potential in the extension may also have generated balloon-like platelets, as indicated by our TEM illustrations. With this theory in mind, it is suggested that the former subpopulation mainly serves as an area for coagulation assembly, participates in the retraction of fibrin clots, and shows a “closed “ GPIIb/IIIa [45], which may explain that these platelets, despite being considered procoagulant, do not have a positive influence regarding clot stability in vitro. Although the DMSO-free platelets generally showed reduced MCF and glycoproteins, slightly beyond the reduction already seen with the standard DMSO method, this difference did not seem to be of such an extent that hemostatic efficiency in vitro could not be demonstrated. It is important, however, that the coagulation capacity in vitro was significantly improved with respect to DMSO-free platelets when using CRF.

In previous studies on DMSO platelets, as well as in the current study, impaired clot stability compared to fresh platelets was shown [12,32]. As shown previously, the MCF was reduced, but by reconstituting the DMSO-free concentrates in less plasma, the platelet concentration could be increased and the MCF increased, creating a more stable clot in vitro. The relevance of this in vivo, when giving a platelet transfusion, is, however, unknown. In contrast, it is suggested that at the high conventional liquid stored platelet concentrations, the association of MCF and platelet concentration is leveled out [48]. Thus, it can be speculated that with an increase in the concentration of cryopreserved platelets, most of these platelets would be PS-positive and therefore likely to contribute to a procoagulant surface.

In accordance with recommendations, the desired DMSO concentration in the final product should not exceed 1% and must therefore be diluted in 200 mL of plasma, while, for the DMSO-free platelets, such precautions are not necessary, and this may be beneficial as our results showed a clear in vitro improvement regarding coagulation when the platelet concentration was increased. Since saline is non-toxic, freezing DMSO-free units in larger volumes without a centrifugation step would hypothetically offer the possibility of reducing the complexity of the process, both pre- and post-freeze/thaw, but the logistical advantages, especially regarding transport to smaller hospitals, of freezing small volumes were of greater importance in choosing the presented study design.

Controlled-rate freezing is favorable to minimize the risk of intracellular ice formation, which therefore may be an advantage [16,49,50]. However, we have previously shown that CRF does not reduce cell damage in platelets cryopreserved with DMSO [12]. In contrast, during DMSO-free freezing, the MMP of the platelets increased on average by 16% and coagulation evaluated in ROTEM was improved when using CRF. This indicates that optimizing the freezing rate method is important in DMSO-free freezing, to optimize cell function. 

One hypothesis about the nature of intracellular ice formation is proposed in which the osmotically driven water efflux that occurs in cells during freezing (caused by the increased osmotic pressure of the extracellular solution in the presence of ice) is viewed as the agent responsible for producing a rupture in the plasma membrane, thus allowing extracellular ice to propagate into the cytoplasm [51]. The pressure that develops during freezing due to water flux is found to be sufficient to cause a rupture in the plasma membrane and the theory gives an accurate description of the phenomenology of intracellular ice formation. However, the outcomes of our experiments indicate that intracellular ice formation is prevented in the presence of a high volume of isotonic saline, hypothetically by contributing to the osmosis effect, while the protective effect on the cell membrane regarding harmful extracellular ice crystallization remains unclear. As intracellular ice formation, as proposed, occurs in cells during freezing but not in a frozen state, the effect of time at −80 °C may be negligible. However, this must be tested in further studies with an extended storage time on such platelets.

A limitation of our study is that it was not always clear what the various alterations in receptor complexes signified. In this setup, it was not possible to determine whether the altered expression of different receptor complexes generally occurred on all cells or reflected a specific fraction of cells—for instance, if the observed change was, hypothetically, directly linked to the aggregating or the procoagulant fraction. Potential inclusions of other subpopulations, as recently described [37,52], may also have played a role in contributing to the complexity. 

In summary, this study has shown, for the first time, that it is possible to freeze platelets in saline in the absence of a CPA. Further studies are needed to fully understand the mechanisms involved. We have shown that DMSO-free cryopreserved platelets are viable and hemostatically active in vitro, which needs to be confirmed in future in vivo studies.

## 4. Materials and Methods

### 4.1. Experimental Overview: Cryopreservation 

**Figure 6:**
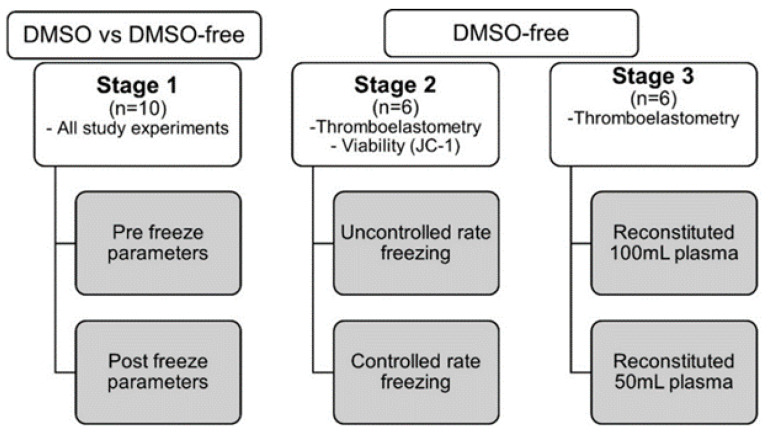
Schematic overview of the study. The figure displays the study, which was divided into three stages. Stage 1, comparing DMSO versus DMSO-free cryopreservation, with measurements taken pre- and post-freezing. All cryopreserved platelets in stage 1 were reconstituted in 200 mL plasma. Stage 2, comparing DMSO-free cryopreserved platelets frozen by uncontrolled (UCRF) or controlled-rate freezing (CRF). All cryopreserved platelets in stage 2 were reconstituted in 200 mL plasma. Stage 3, comparing the hemostatic function of DMSO-free cryopreserved platelets (UCRF) reconstituted in 100 mL or 50 mL plasma.

In the first experiment, the cryopreserved platelets were prepared from double-dose platelet concentrates (*n* = 10) produced from pools of eight ABO-identical buffy coats in additive solution (SSP+), as previously described [53]. Each double dose of platelet concentrate was then equally divided into two separate freezing bags (Macopharma), using a sterile connection device (Terumo BCT, Lakewood, CO, USA). Additionally, a small sample (10 mL) was sterilely collected from the double-dose to determine the baseline in vitro parameters. Each production line (*n* = 10) of the study was then prepared accordingly. Freezing medium comprising either 25% DMSO/NaCl (9 mg/mL) (50 mL) or NaCl (9 mg/mL) (100 mL) was sterilely docked and then added to the respective platelet concentrate from the same double dose. The platelet concentrates were centrifugated for 10 min at 1200, and nearly all the supernatant was removed, leaving approximately 0.5 mL freezing medium in approximately 10 mL of platelet suspension, resulting in a final concentration of 5% DMSO/NaCl or 10% NaCl. The platelet concentrates were immediately placed in metal boxes (Ninolab) and frozen at −80 °C with fast uncontrolled freezing. Cryopreserved units were then stored at −80 °C for 70–119 days, subsequently thawed, and resuspended as recently described [30] in 200 mL fresh plasma. After thawing, all units were kept at room temperature for one hour before analysis (Figure 6).

In a second experiment, the double-dose platelet concentrate (*n* = 6) was cryopreserved in NaCl (9 mg/mL) (100 mL) using either fast uncontrolled freezing or controlled-rate freezing. Controlled-rate freezing was performed with a liquid nitrogen freezer, the Planer Kryo 560-16 (Planer, Middlesex), with a set temperature program from +4 °C to −90 °C, as described [12]. Cryopreserved units were then stored at −80 °C until thawed and resuspended as mentioned. Furthermore, in a third experiment, the double-dose platelet concentrate (*n* = 6) was cryopreserved according to the above-described procedure with NaCl (9 mg/mL) (100 mL) as the freezing medium, and later thawed in either 50 mL or 100 mL blood-group-matched plasma (Figure 6).

### 4.2. Intra- and Extracellular Metabolic Parameters

In vitro metabolic parameters and recovery were analyzed before and after freezing. Platelet counts and mean platelet volumes were measured using the CA 620 Cellguard (Boule Medical, Stockholm, Sweden). Routine blood gas equipment (ABL 800, Radiometer Medical ApS, Copenhagen, Denmark) was used to analyze the pH (at 37 °C), glucose, lactate, and bicarbonate together with electrolytes (cNa+, cK+, and cCl−). The total adenosine triphosphate (ATP) concentration was measured using the ATP kit SL 144-401 (BioThema Luminescent Assays). Duplicates of platelet samples were diluted 1:10 in 0.6% trichloroacetic acid (0.73 M TCA, H_2_O Sigma Aldrich, Stockholm, Sweden) and incubated for 15 min on ice. Thereafter, the samples were further diluted 1:81 in Tris–EDTA buffer (0.1 M Tris[hydroxymethyl] aminomethane, 2 mM EDTA, adjusted to pH 7.75 with acetic acid) and directly frozen at −60 °C. All frozen samples were then analyzed in one batch, with triplicates of each sample. Frozen samples were thawed at room temperature and added to a 96-well microplate (Biothema) with 120 μL Tris–EDTA buffer. A total of 40 μL ATP Reagent SL (D-luciferin, luciferase, magnesium ions, inorganic pyrophosphate, and bovine serum albumin) was added to all samples. Light emission corresponding to sample ATP levels (μmol/1011 platelets) was measured with a luminometer (Orion Microplate, Berthold Detection Systems GmbH, Pforzheim, Germany).

### 4.3. Flow Cytometry

Flow cytometry was used to analyze the expression of a variety of platelet markers, such as P-selectin with PE-conjugated CD62P (Beckman Coulter, Brea, CA, USA); GPIb with FITC-conjugated CD42b (Beckman Coulter); platelet endothelial cell adhesion molecule with PECAM-1 (Sigma-Aldrich, St. Louis, MI, USA); GPIIb with PE-conjugated CD61 (Beckman Coulter, Indianapolis, IN, USA); platelet collagen receptor with FITC-conjugated GPVI (Pharmingen BD Biosciences, San Jose, CA, USA); and phosphatidylserine with Annexin V (BD Biosciences, Erembodegerm, Belgium). The conformational epitope on the GPIIb/IIIa complex of activated platelets was assessed before and after stimulation with collagen (50 μg/mL, Sigma Aldrich) and ADP (10 μM, Sigma Aldrich) using the FITC-conjugated monoclonal antibody PAC-1 (IgM, Becton Dickinson, Franklin Lakes, NJ, USA). Changes in the platelet mitochondria transmembrane potential (Δψ) were assessed with the mitochondrial permeability transition detection kit MitoPT JC-1 (Immuno-Chemistry Technologies, LCC, Bloomington, MN, USA). All flow cytometric tests were executed using a CytoFLEX Flow Cytometer (Beckman Coulter Life Sciences, Bromma, Sweden) as previously described [11,12,53].

Platelet microparticles (PMPs) were analyzed directly from the platelet units (fresh, DMSO, and DMSO-free) without centrifugation, to minimize sample manipulation. Beads of standardized 0.6, 1.0, and 3.0 μmol/L (Biocytex, Marseille, France) were used to set the gating scale for the forward light scatter to distinguish the microparticles. PMPs were defined as being less than 1 µm, based on the binding of platelet glycoproteins GPIIb/IIIa CD61 PE-conjugated (Beckman Coulter, Indianapolis, IN, USA), dual-stained with Annexin-V FITC-conjugated (BD Biosciences, Erembodegerm, Belgium), diluted in Annexin V binding buffer (BD Biosciences, Erembodegerm, Belgium) [13]. The proportion (%) of PMPs was then calculated from the total number of 10,000 gated events, analyzed on a CytoFLEX Flow Cytometer (Beckman Coulter Life Sciences).

### 4.4. Thromboelastometry 

Thromboelastometry using a ROTEM delta 3000 (TEM International, GmbH, Munich, Germany) was performed to assess the hemostatic function of the platelets. To begin, cryopreserved DMSO and DMSO-free (*n* = 10) were diluted in aliquoted fresh frozen AB plasma to a concentration of 200 × 10^9^ viable cells /L according to the expression of JC-1+% of total cells. Diluted samples were then analyzed on the instrument according to the manufacturer’s instructions, with the EXTEM parameter as the readout. 

Subsequent analyses were carried out with DMSO-free platelets cryopreserved using either controlled-rate freezing or fast uncontrolled-rate freezing (*n* = 6). Samples from the two groups were directly analyzed on the instrument without any further dilution. Additionally, tests were performed on cryopreserved DMSO-free platelets (*n* = 6) reconstituted in either 100 mL or 50 mL fresh blood-group-compatible plasma. Samples from each bag were directly analyzed in parallel, with one sample diluted to a concentration of 200 × 10^9^ cells/L.

### 4.5. Transmission Electron Microscopy

For transmission electron microscopy (TEM), platelets were fixed in 2% glutaraldehyde in 0.1 M phosphate buffer, pH 7.4, at room temperature, for 30 min, followed by overnight fixation at 4 C. After fixation, platelets were rinsed in 0.1 M phosphate buffer pH 7.4. The cells were gently centrifuged to a pellet and postfixed in 2% osmium tetroxide in 0.1 M phosphate buffer, pH 7.4, at 4 C, for 2 h, stepwise dehydrated in ethanol followed by acetone, and finally resin embedded in LX-112 (Ladd). The ultrathin sections were stained with uranyl acetate followed by lead citrate and examined in a Hitachi HT7700 electron microscope (Hitachi High-Tech) operated at 80 kV. Digital images were obtained using a 2kx2k Veleta digital camera (OSiS). Digital images were included to illustrate the appearance of ultrastructural alterations in platelets pre-freezing and post-thawing.

### 4.6. Statistics 

The mean values, standard deviations, and error bars 95% CI are given if not further specified. A paired *t*-test was applied to determine the statistical significance (*p* < 0.05) between the DMSO and DMSO-free cryopreserved platelet derivates. Further, *t*-tests were performed between CRF and UCRF for DMSO-free products. Additionally, paired *t*-tests were performed between groups of DMSO-free platelet products reconstituted in either 50 mL or 100 mL plasma. ANOVA was performed to investigate differences in microparticle amounts between fresh, cryopreserved DMSO, and cryopreserved DMSO-free platelets. All the statistical analyses were carried out using IBM SPSS statistics (version 28.0.1.1 (14)). 

## Figures and Tables

**Table 1:** Extra- and intracellular metabolic parameters before and after cryopreservation. The table displays values from fresh platelets (double-dose buffy coat platelets pre-freezing) and post-thaw DMSO-containing cryopreserved platelets compared to post-thaw DMSO-free cryopreserved platelets. Data are presented as mean  ±  SD of *n* = 10. A two-tailed *t*-test was performed between the DMSO and DMSO-free groups, defined as ** *p* < 0.01, and *** *p* < 0.001.

	Pre-Freeze Fresh Platelets	Post-Thaw DMSO	Post-Thaw DMSO-Free	*p*-Value
**Mean platelet volume** (fL)	9.61 ± 0.30	9.92 ± 0.50	10.25 ± 0.96	0.155
**pH** (37 °C)	6.94 ± 0.05	7.09 ± 0.07	7.11 ± 0.07	<0.001 ***
**pCO2** (kPa)	5.96 ± 0.76	8.29 ± 0.71	7.85 ± 0.69	<0.001 ***
**pO2** (kPa)	16.79 ± 1.78	16.52 ± 1.13	20.42 ± 0.66	<0.001 ***
**Glucose** (mmol/L)	8.00 ± 0.59	16.29 ± 3.98	16.40 ± 3.86	0.335
**Lactate** (mmol/L)	7.65 ± 1.05	2.89 ± 0.75	2.64 ± 0.67	<0.001 ***
**Bicarbonate** (calculated)	8.98 ± 0.57	18.09 ± 2.19	17.86 ± 2.23	0.003 **
**cK^+^** (mmol/L)	4.04 ± 0.08	4.29 ± 0.21	4.32 ± 0.23	0.394
**cNa^+^** (mmol/L)	159.90 ± 1.10	156.10 ± 2.33	154.50 ± 1.35	0.005 **
**cCl^−^** (mmol/L)	79.90 ± 2.13	84.30 ± 1.57	84.90 ± 1.91	0.81
**ATP** (μmol/10^11^ PLTs)	6.45 ± 1.16	4.03 ± 1.39	2.79 ± 1.54	0.01 **

## Data Availability

Raw data are currently unavailable due to Karolinska policy restrictions.
